# The superiority of conditioned medium derived from rapidly expanded mesenchymal stem cells for neural repair

**DOI:** 10.1186/s13287-019-1491-7

**Published:** 2019-12-16

**Authors:** Ya-Tzu Chen, May-Jywan Tsai, Nini Hsieh, Ming-Jei Lo, Meng-Jen Lee, Henrich Cheng, Wen-Cheng Huang

**Affiliations:** 1 Institute of Pharmacology, School of Medicine, National Yang-Ming University, Taipei, 11221 Taiwan; 2 Neural Regeneration Laboratory, Department of Neurosurgery, Neurological Institute, Taipei Veterans General Hospital, Taipei, 11217 Taiwan; 3 Department of Applied Chemistry, Chaoyang University of Technology, Taichung, Taiwan; 4 Department of Medicine, National Yang-Ming University, Taipei, 11221 Taiwan; 5 Department of Neurosurgery, Neurological Institute, Taipei Veterans General Hospital, Taipei, Taiwan; 6 Division of Neural Regeneration and Repair, Neurological Institute, Taipei Veterans General Hospital, Taipei, Taiwan

**Keywords:** Spinal cord injury, Mesenchymal stem cell, Xeno-free medium, Secreted factors

## Abstract

**Background:**

Spinal cord injury (SCI) is a complex and severe neurological condition. Mesenchymal stem cells (MSCs) and their secreted factors show promising potential for regenerative medicine. Many studies have investigated MSC expansion efficacy of all kinds of culture medium formulations, such as growth factor-supplemented or xeno-free medium. However, very few studies have focused on the potential of human MSC (hMSC) culture medium formulations for injured spinal cord repair. In this study, we investigated the effect of hMSC-conditioned medium supplemented with bFGF, EGF, and patient plasma, namely, neural regeneration laboratory medium (NRLM), on SCI in vitro and in vivo.

**Methods:**

Commercial and patient bone marrow hMSCs were obtained for cultivation in standard medium and NRLM separately. Several characteristics, including CD marker expression, differentiation, and growth curves, were compared between MSCs cultured in standard medium and NRLM. Additionally, we investigated the effect of the conditioned medium (referred to as NRLM-CM) on neural repair, including inflammation inhibition, neurite regeneration, and spinal cord injury (SCI), and used a coculture system to detect the neural repair function of NRLM-MSCs.

**Results:**

Compared to standard culture medium, NRLM-CM had superior in inflammation reduction and neurite regeneration effects in vitro and improved functional restoration in SCI rats in vivo. In comparison with standard culture medium MSCs, NRLM-MSCs proliferated faster regardless of the age of the donor. NRLM-MSCs also showed increased adipose differentiative potential and reduced CD90 expression. Both types of hMSC CM effectively enhanced injured neurite outgrowth and protected against H_2_O_2_ toxicity in spinal cord neuron cultures. Cytokine arrays performed in hMSC-CM further revealed the presence of at least 120 proteins. Among these proteins, 6 demonstrated significantly increased expression in NRLM-CM: adiponectin (Acrp30), angiogenin (ANG), HGF, NAP-2, uPAR, and IGFBP2.

**Conclusions:**

The NRLM culture system provides rapid expansion effects and functional hMSCs. The superiority of the derived conditioned medium on neural repair shows potential for future clinical applications.

## Introduction

Spinal cord injury (SCI) is a debilitating and complex neurological condition. The primary damage caused by external forces is followed by a secondary injury cascade involving oxidative stress, excitotoxicity, metabolic changes, inflammatory processes, and necrosis or apoptosis; the induced gliosis and fibrosis produce a challenging environment for axonal regrowth [1–5]. In general, axons fail to regenerate, and neurologic recovery is prevented by the poor intrinsic regeneration capacity of neurons and by the presence of growth inhibitors in the adult spinal cord. Therefore, there are several strategies in SCI therapy to improve the hostile injury environment, including supplying trophic factors for cell protection [6, 7], chondroitinase ABC for proteoglycan degradation to decrease the concentration of inhibitory molecules [8], and Decoy 3/minocycline to reduce inflammation [9, 10]. The other strategies involve the gene-mediated enhancement of intrinsic neuronal regrowth machinery after injury, such as the modulation of the PTEN/mTOR pathways [3, 11, 12], and using cell therapy to replace the dead or damaged cells, such as neural stem cells (NSCs) or mesenchymal stem cell (MSCs) [13, 14].

MSCs are progenitor cells from the mesoderm and are also accessible from the bone marrow, adipose tissue, and placenta for culture [15]. MSCs secrete many kinds of neurotrophic factors [16, 17] and display poor immunogenicity [18]. MSCs transdifferentiate into neural lineage cells in appropriate experimental conditions [19, 20] and modulate the immunoreactivity to reduce inflammation [21]. MSC transplantation has been shown to improve neurological and movement functions in rodents after SCI [22]. In recent years, MSC transplantation has been used in clinical trials to treat SCI and has been shown to improve the total AIS grade with no serious adverse effects [23, 24]. MSC transplantation has unique effects in spinal cord repair via the inhibition of inflammatory cell activation, reconstitution of local blood supply systems, and cell protection, which are produced by the MSC paracrine effect [14, 24]. Some pre-induced MSCs differentiate into neural lineage cells after transplantation, but the evidence for naive MSC differentiation into functional neural lineage cells in vivo is weak [24, 25]. Therefore, MSC-conditioned medium (MSC-CM) is considered an attractive candidate for stem cell-based therapeutic applications. MSC-CM has been reported to increase neuronal survival in glutamate excitotoxicity and oxygen-glucose deprivation (OGD) environments [26, 27] and promote hindlimb functional restoration in SCI rats [28–30]. Factors secreted by MSCs could contribute to immunoregulation and regeneration. MSCs behave as extracorporeal bioreactors and secrete bioactive factors into the CM that may become a novel therapeutic tool for clinical use in CNS injury [30–32].

The yield and efficacy of MSC-CM are essential issues for future clinical applications. The use of growth factor supplements such as bFGF and EGF in MSC cultures could have marked effects on MSC proliferation [33–36]. However, most studies have focused on the changes in the expansion and characteristics of MSCs in different culture medium formulations, but there has been no further clarification of the therapeutic effects of MSC-CM formulations.

In the present study, human MSCs from commercially obtained/clinical bone marrow samples were cultured in bFGF- and EGF-supplemented medium containing fetal bovine serum (FBS)/patient’s plasma, namely, neural regeneration laboratory medium (NRLM), for rapid ex vivo MSC expansion. Furthermore, we investigated the therapeutic effect of the conditional medium from MSCs cultivated in NRLM (referred to as NRLM-CM) on SCI in vitro and in vivo. NRLM-CM displayed superior inflammation reduction and neurite regeneration effects in vitro and enhanced functional restoration in SCI rats. NRLM was also successfully used to culture MSCs derived from clinical patient bone marrow samples.

## Methods

### Cells, reagent, and antibodies

BV2 microglia were a gift from Dr. Shiao [37]. One human MSC clone was purchased from Lonza (lot no: PT-2501, Basel, Switzerland). Millicell culture inserts were purchased from Millipore (Watford, UK). A TUNEL assay kit was from Promega. Cell culture medium, FBS, serum-free supplements, and antibiotics were purchased from Invitrogen (Carlsbad, CA, USA). Tissue culture plastics were obtained from BD Biosciences (San Jose, CA, USA). Primary antibodies were rabbit and mouse anti-neuronal class III β-tubulin (clone TUJ-1, Covance, NJ, USA), rabbit anti-GFAP (DakoCytomation, Ely, UK), mouse anti-5′-bromo-2′-deoxyuridine (BrdU; Millipore, Watford, UK), and anti-GAP-43 (Invitrogen, CA, USA). Unless stated otherwise, all other chemicals were purchased from Sigma-Aldrich Co.

### Human sample collection

The procedure for human sample collection followed the guidelines approved by our hospital’s ethics committee (Institutional Review Board IRB 2012-04-049B). Bone marrow aspirates were collected from the spine of 30 patients with cervical, thoracic, or lumbar diseases undergoing spinal fusion surgery at the Taipei Veterans General Hospital (Taipei, Taiwan). In addition, peripheral blood was collected from consenting patients. Patient plasma was collected in a heparin-coated tube and subsequently spun by centrifugation (400×*g* for 10 min) to obtain the supernatant (plasma). Each patient plasma sample was individually stored at − 20 °C for the subsequent culture medium supplementation.

### Preparation of human bone marrow mesenchymal stem cells (BM-MSCs) and MSC-conditioned medium (MSC-CM)

The bone marrow aspirate was layered onto Ficoll-Paque PLUS solution (Amersham Biosciences, Fairfield, CT) and spun (400×*g* for 30 min) to deplete the red blood cells, platelets, and plasma. Ficoll-fractionated mononuclear cells were collected from the gradient interphase and washed twice with phosphate-buffered saline (PBS; Sigma). The cells were then seeded in two 25T flasks with different growth media: [1] MSC growth medium including 10% FBS (MSCGM™; Lonza/Cambrex, Basel, Switzerland), named MSCGM or [2] DMEM/F12 supplemented with B27 supplement (Invitrogen), 20 ng/ml bFGF (R&D), 20 ng/ml EGF (Invitrogen), and 10% patient plasma (named NRL medium, NRLM) at 37 °C in a water-saturated atmosphere of 5% CO_2_/95% air. All growth medium was supplemented with 1% penicillin/streptomycin (Invitrogen). Approximately 3–5 days after cell seeding, cultures developed MSC colonies. At 10–14 days, confluent cells were subcultured and expanded. The CM was collected from two to three passages of two kinds of cultivated hMSCs. Briefly, confluent MSCs were lifted by trypsinization and re-seeded at a density of 10^6^ cells per flask (75 cm^2^). The next day, the cells were washed twice with PBS to remove any residual factors and serum. Cultures were re-fed with 12 ml of serum-free DMEM and incubated for 48 h. The CM was collected and filtrated through a 0.2-μm filter to remove cellular debris. The filtrate was further concentrated ~ 30-fold by a centrifugal filter device (3 kDa cutoff; Amicon Ultra, Millipore) and stored frozen at − 80 °C.

### Characterization of cultivated human BM-MSCs and MSC-CMs

The specific surface markers of isolated and expanded bone marrow cells were detected at the second and third passages by flow cytometric analysis. Human MSCs were harvested by treatment with 0.25% trypsin (Gibco). The cells were stained for 1 h at room temperature (RT) with phycoerythrin- (PE-) conjugated antibodies for cell surface markers, including CD34 (hematopoietic lineage early marker; Beckman, IM1781U), CD45 (Beckman, IM2078U), CD90 (Ty-1; Beckman, IM1840U), CD44 (Beckman, A32537), CD166 (Beckman, A22361), CD29 (integrin; BD Biosciences, 555443), CD73 (BD Biosciences, 550257), and CD105 (BD Biosciences, 560839). The stained cells were analyzed by a fluorescence-activated cell sorter (Canto II; BD Biosciences) using a 575-nm bandpass filter for red PE fluorescence. The proliferation of the two kinds of cells was investigated by pulsing subconfluent cells with 25 μM 5-bromo-2′-deoxyuridine (BrdU; Sigma) for 6 h. The cells were then fixed and immunostained with anti-BrdU. Cells were also lifted by trypsinization and counted with a hemocytometer to allow the effective discrimination of live from dead cells using trypan blue exclusion at culture days 3, 5, and 7. The differentiation potential of the cultured MSCs was examined using commercial adipogenesis, chondrogenesis, and osteogenesis kits (Mesenchymal Stem Cell Adipogenesis Kit, Chemicon SCR020; StemPro® Chondrogenesis Differentiation Kit, Gibco A10071 01; StemPro® Osteogenesis Differentiation Kit, Gibco A10072-01) according to the manufacturer’s instructions. We analyzed the expression of a panel of proteins in MSC-CM using a membrane-based human cytokine antibody array kit (RayBiotech, Norcross, GA). The relative expression levels of 120 soluble human proteins were determined. The black spots revealed were analyzed using an ImageJ protein array analyzer. The intensity of each spot was normalized to that of a positive control spot on each membrane. The data were displayed on a multicolor heat map using GraphPad Prism.

### BV2 culture and nitric oxide assays

We used lipopolysaccharide (LPS)-stimulated microglia BV2 cultures to assess the anti-inflammatory effect of MSC-CM. LPS is a cell wall component of gram-negative bacteria and a powerful immunostimulatory factor. BV2 microglial cells were first challenged with 0.5 μg/ml LPS in DMEM containing 2% FBS for 24 h. Various doses of MSC-CM (30×) ranging from 0 to 2% (v/v) were then added, and the cultures were further incubated for 24 h. At the endpoint, the CM was collected for nitric oxide (NO) measurement. The production of NO was assayed as the accumulation of nitrite in the medium using a colorimetric reaction with Griess reagent (1% sulfanilamide/0.1% naphthyl ethylenediamine dihydrochloride/2% phosphoric acid), as previously described [38]. To assess the axon regeneration potential of MSCs, we used transwell cocultures.

### Rat spinal cord mixed neuron-glial cultures

Mixed neuronal-glial cell cultures were prepared from the spinal cord regions of embryonic Sprague-Dawley rats at gestation days 15–17. We modified the methods described in previous articles [38, 39] to culture spinal neurons and glia. Briefly, fetal spinal cords were physically dissociated with trituration and filtration through a 70-μm cell strainer. The cell filtrates were collected and seeded on poly-l-lysine-coated 1-μm PET hanging transwell inserts (Millipore, Watford, UK) in 24-well plates at a density of 6 × 10^5^ cells/cm^2^ and maintained in high-glucose DMEM (h-DMEM) containing 10% FBS and 1% penicillin-streptomycin at 37 °C in a water-saturated atmosphere of 5% CO_2_ and 95% mixed air. After incubation overnight, the culture medium was replaced with h-DMEM supplemented with N2 (Invitrogen, for serum-free conditions). The cells were maintained in the above medium for 3–5 days for follow-up axonal regeneration and neuroprotection analysis.

### Neural regeneration and neuroprotection assays

For the assessment of neural regeneration, mixed neuronal/glial cell cultures were seeded on 1-μm PET hanging transwell inserts inside a 24-well plate and maintained for 3–5 days. Axons passed through the porous membrane and ran parallel to the cell body layer. The neurites extending through the membrane were wiped off, mimicking axonal injury. The injured cell cultures on the transwell were then treated with CM or cocultured with hMSCs grown as attached cells. The analysis of axonal regrowth was performed 5 days after coculture. Cells were harvested by fixation with 4% paraformaldehyde and processed for immunostaining against GAP-43, an axonal regeneration marker. The cell bodies inside the transwell were removed, leaving the extending neurites intact on the other side of the transwell. The insert membrane was cut and transferred to slides for microscopic observation. Three groups of patient samples were tested, with three replicates performed per culture. Three fields of view were randomly selected to analyze the intensity of GAP-43 expression. The GAP-43-positive signal densitometry measurements were taken by using ImageJ software after the removal of the background signal, and the signals were normalized by field. To analyze neuroprotection, spinal cord neuron-glial cultures were seeded on coverslips in N_2_-supplemented h-DMEM for 3–5 days. The coverslips were transferred to 24-well plates on which hMSCs had been seeded. The cocultures were then treated with 120 μM H_2_O_2_ to induce oxidative stress. Twenty-four hours after treatment, the cells were fixed, and apoptotic cells were detected using the TUNEL assay (DeadEnd™ Fluorometric TUNEL System, Promega). The images (× 200 magnification) of TUNEL-positive signal cells were quantified by the ImageJ plus cell counter module. Each patient MSC sample was tested three times. Three fields of view were randomly selected to count the number of TUNEL signals and the number of nuclei.

### Intrathecal catheter implantation

Adult female Sprague-Dawley rats (approximately 250–270 g) were used for the experiment. All experimental procedures were carefully reviewed and approved by the Animal Studies Subcommittee of Taipei Veterans Hospital (IACUC 2013-187). The rats were anesthetized with 1–2% isoflurane, and the body temperature was maintained at 37 °C during surgery with a thermic blanket. The skin was cut by a scalpel, and the third and fourth quarters of the lumbar vertebrae were revealed. The connective tissue in the L4–5 interspace was removed, and then the spinal cord was exposed. A homemade intrathecal catheter and an injection cap were filled with normal saline, and then the intrathecal catheter was slowly inserted into the spinal cord dura space from the L4–5 interspace. The injection cap was fixed to the back muscle using a nylon suture. The skin was then sutured, and the whole injection cap was enclosed within the skin. The animals were awoken after being returned to their cages. The animals were observed for 5 days to ensure the hindlimb movements were normal.

### Spinal cord transection injury and intrathecal delivery of MSC-CM

After the confirmation of normal hindlimb movement, the rats received a T8 spine total laminectomy for the exposure of the spinal cord and complete transection by microscissors. To confirm the complete transection of the spinal cord, we used microscopic forceps to tear the remaining dura along the spine once. Avoiding blood clot formation in the catheter, 1 ml of saline was slowly injected into the injection cap after complete transection to test the transition. Saline outflow was observed at the injury site. In the case of an obstruction, the catheter was exchanged, or the animals were not included in the study. Following injury, the incision was closed and sutured. The urine was manually expressed twice daily. Prophylactic antibiotics (0.5 ml/day, subcutaneous, Borgal 6.5%) were administered daily for three consecutive days. Following T8 complete transection surgery, the SCI rats were randomly divided into three groups and intrathecally administered saline, MSCGM-CM, or NRLM-CM. The CM (15 μl) was given first on the surgery day and then every week for eight consecutive weeks through injection cap under inhalation anesthesia.

### Behavioral evaluation and tissue preparation

All experimental rats were evaluated for hindlimb behavior by the Basso, Beattie, and Bresnahan (BBB) open field score every week after surgery. Rat behavior was observed for 5 min in an open space of 85 cm in diameter. In this study, the examiner was blinded to each group. In this evaluation of motor performance, 0 represents no locomotion and 21 represents normal motor function. After the last behavioral evaluation (8 weeks after surgery), the rats received an overdose of sodium pentobarbital and were perfused intravascularly with saline and 4% paraformaldehyde in PBS. The spinal cords were collected and placed in 4% paraformaldehyde for postfixation. The next day, the cords were placed in 15% sucrose solution overnight and finally placed in 30% sucrose solution for dehydration. After dehydration, the spinal cords were embedded in optimal cutting temperature (OCT) compound, longitudinally sectioned (20 μm thick), and placed on slides.

### Immunostaining and tissue quantification

Cultured cells were fixed with 4% paraformaldehyde for 15 min and then washed three times with PBS. The tissue section slides were also washed with PBS to remove the OCT compound. The cells and slides were further permeabilized with 0.1% Triton X-100, blocked with 1% bovine serum albumin for 30 min at room temperature, and incubated with primary antibodies overnight at 4 °C, followed by the respective fluorescently tagged secondary antibodies for 1 h at RT. Primary antibodies included mouse anti-GAP-43 (1:500; Invitrogen, 33-5000), rabbit anti-GFAP (1:1000; DakoCytomation, Z0334), and mouse anti-βIII-tubulin (1:500; Covance; MMS-435P). The secondary antibodies included Alexa 594-conjugated donkey anti-rabbit IgG and Alexa 488 conjugated donkey anti-mouse IgG (1:500; Thermo Fisher, A21207 and A21202). Before being blocked with 1% bovine serum albumin, the cells in the BrdU incorporation assay were treated with 2 N HCl for 5 min and then rapidly washed with PBS. The cells were treated according to standard immunocytochemistry protocols. Antibodies, dilutions, and incubations were as follows: mouse anti-BrdU antibody (Millipore, MAB3222) 1:250 at 4 °C overnight and a biotin-conjugated anti-mouse IgG antibody (Vector, BA2001) 1:500 for 1 h at RT. The ABC complex system (Vector, PK-6100) was used to amplify the signal. BAD was used as a substrate to react with peroxides. Images of cultured cells were obtained with a fluorescence microscope equipped with fluorescence optics and a CCD camera. We followed the methods described in a previous article [8] to quantify axon regeneration. The whole spinal cord merged images were exported to ImageJ software (NIH Image, USA) to measure the area of the βIII-tubulin-positive signal in the lesion epicenter area (GFAP-negative area) in two midline longitudinal sections as follows. The selected signal intensity threshold was 20 for βIII-tubulin immunoreactivity. The βIII-tubulin-positive areas with intensities higher than this threshold were calculated and divided by the total selected area between the rostral and caudal borders of the lesion area.

### Statistical analysis

Data were expressed as the mean ± standard deviation, and statistical analyses were performed using GraphPad Prism (version 7.0). A two-tailed paired Student’s *t* test was used for comparisons of two conditions. In all other cases, one- or two-way ANOVA tests were used, as indicated in the figure legends. A value of *p* < 0.05 was considered statistically significant.

## Results

### The characteristics of commercially obtained MSCs cultivated in NRL medium

**Fig. 1:**
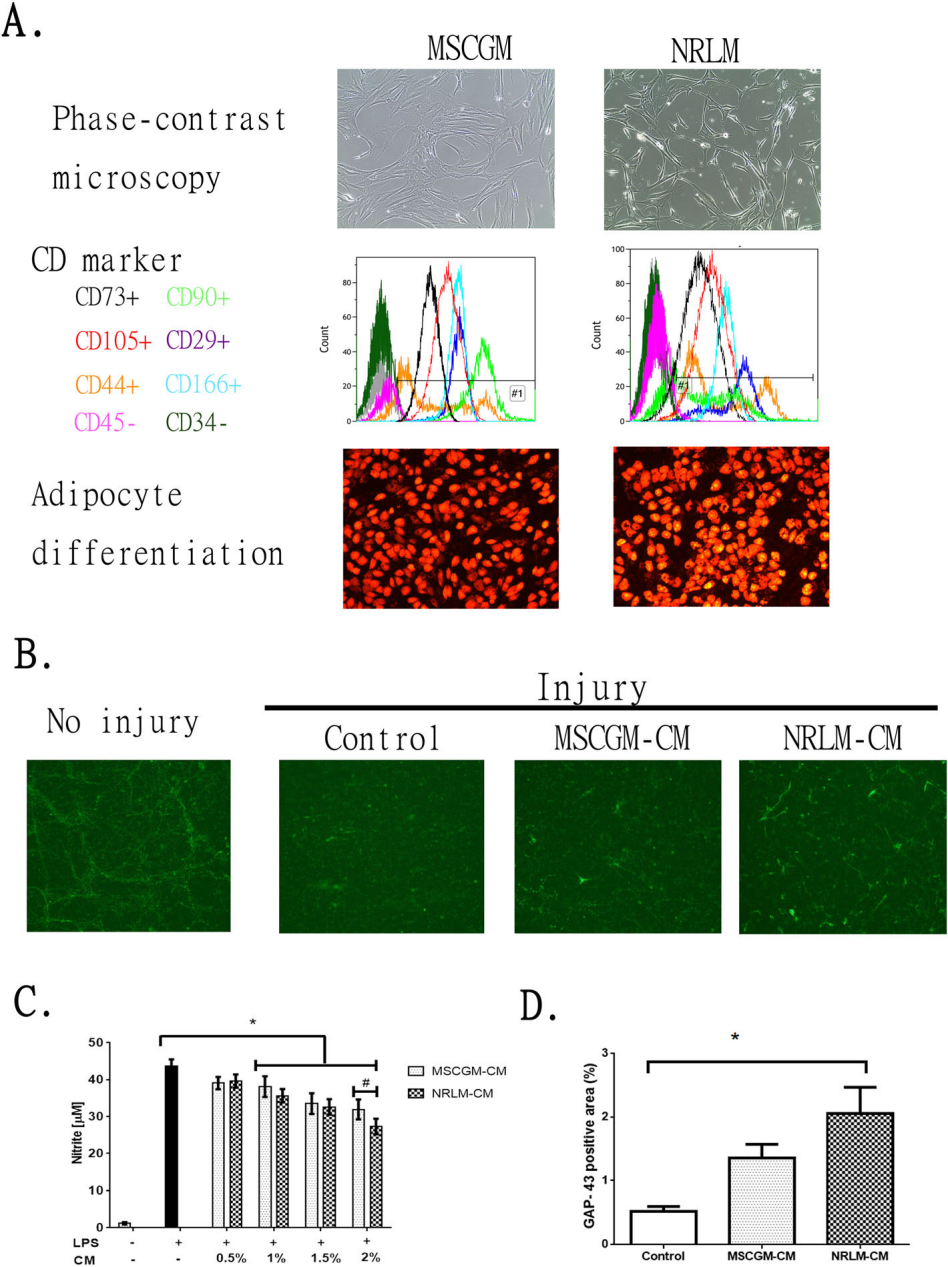
Comparative characterization of commercial mesenchymal stem cells (MSCs) cultured in MSCGM or NRLM and the function of the conditioned medium (CM). **a** MSC characteristics, including morphology (phase-contrast micrograph, upper panel), cell surface expression of CD markers (by flow cytometry, middle panel), and adipocyte differentiation (oil-positive signal after oil staining, lower panel), are shown. **b** The effect of MSCGM-CM and NRLM-CM on the extension of regenerated neurites (GAP-43-positive neurite). **c** Quantitative results from **b**. **d** The effect of MSCGM-CM and NRLM-CM on LPS stimulation in BV2 microglia. Nitrite release in the medium was measured after microglia were treated with LPS in the presence of CM (0–2% v/v). Data represent the mean ± SEM. *p < 0.05 indicates statistical significance using one-way ANOVA with Tukey’s multiple comparisons test

A commercially available human MSC line (lot no. PT-2501), obtained from Lonza Co. (Basel, Switzerland), was first used as a standard culture to evaluate the growth conditions designed in the present study. We confirmed the characteristics of the commercially obtained MSCs cultured in our NRL medium (designated as NRLM-MSCs) by morphology, differentiation potential, and cell surface CD marker expression. NRLM-MSCs possessed a fibroblast-like morphology and the ability to differentiate into adipocytes, as shown in Fig. 1a. Following the protocol described in the MSC Adipogenesis Kit (Chemicon SCR020), we found that MSCs differentiated into adipocytes bearing oil-positive signals (positive AdipoRedTM assay reagent staining, Lonza). NRLM-MSCs expressed CD29, CD44, CD73, CD105, and CD166 but did express the hematopoietic markers CD34 and CD45, similar to the CD marker expression of MSCs maintained in standard MSC growth medium (MSCGM-MSCs). The expression level of CD90 was lower in NRLM-MSCs than in MSCGM-MSCs (Fig. 1a). The effects of CM derived from MSCs cultured in two conditions, NRLM-MSCs and MSCGM-MSCs, were examined in spinal cord neuron cultures and microglia. The CM from NRLM-MSCs and MSCGM-MSCs was named NRLM-CM and MSCGM-CM, respectively.

### Validation of the LPS stimulation reduction and neural regeneration enhancement effects of MSC-CM

By mimicking the status of the inflammatory response and axonal injury after SCI, we tested the effects of MSC-CM on LPS-stimulated microglia and on injury-induced axonal regeneration in spinal cord neuron cultures. LPS is a cell wall component of gram-negative bacteria and a powerful immunostimulatory factor. We previously showed that LPS treatment induces an increase in iNOS expression and NO release in spinal cord cultures and microglia [38]. In this study, we used BV2 cells, immortalized murine microglia, as an in vitro model of LPS stimulation to investigate the effect of MSC-CM on activated microglia. After 24 h of stimulation of microglia with LPS, doses of CM were added to the culture medium, and the microglia were further incubated for 24 h. Figure 1c shows that CM from both MSC cultures significantly decreased LPS-induced nitrite release from BV2 microglia in a dose-dependent manner. At the 2% CM dose, NRLM-CM was more effective at decreasing LPS stimulation than MSCGM-CM (*p* < 0.05). We also designed an in vitro neuronal regeneration model to examine the effects of MSC-CM in neuronal cultures after injury. Rat spinal cord neurons were seeded in 1-μm PET hanging transwell inserts in a 24-well plate and maintained for 3–5 days. Only axons passed through the porous membrane and ran parallel to the cell monolayer. The neurites passing through the membrane were wiped off, mimicking axonal injury. The cells in transwell inserts were then treated with 2% MSC-CM for 5 days. Figure 1b shows that axonal injury reduced the number of GAP-43-positive neurites growing through the transwell membrane. MSC-CM treatment increased GAP-43-positive neurite extension. The GAP-43-positive area percentages among the groups were calculated by ImageJ software, and the quantitative results are shown in Fig. 1d. Treatment with NRLM-CM markedly protected injury-induced GAP-43 neurite regeneration in spinal cord neuron cultures (*p* < 0.05).

### Improvement of hindlimb functional recovery by MSC-CM

**Fig. 2:**
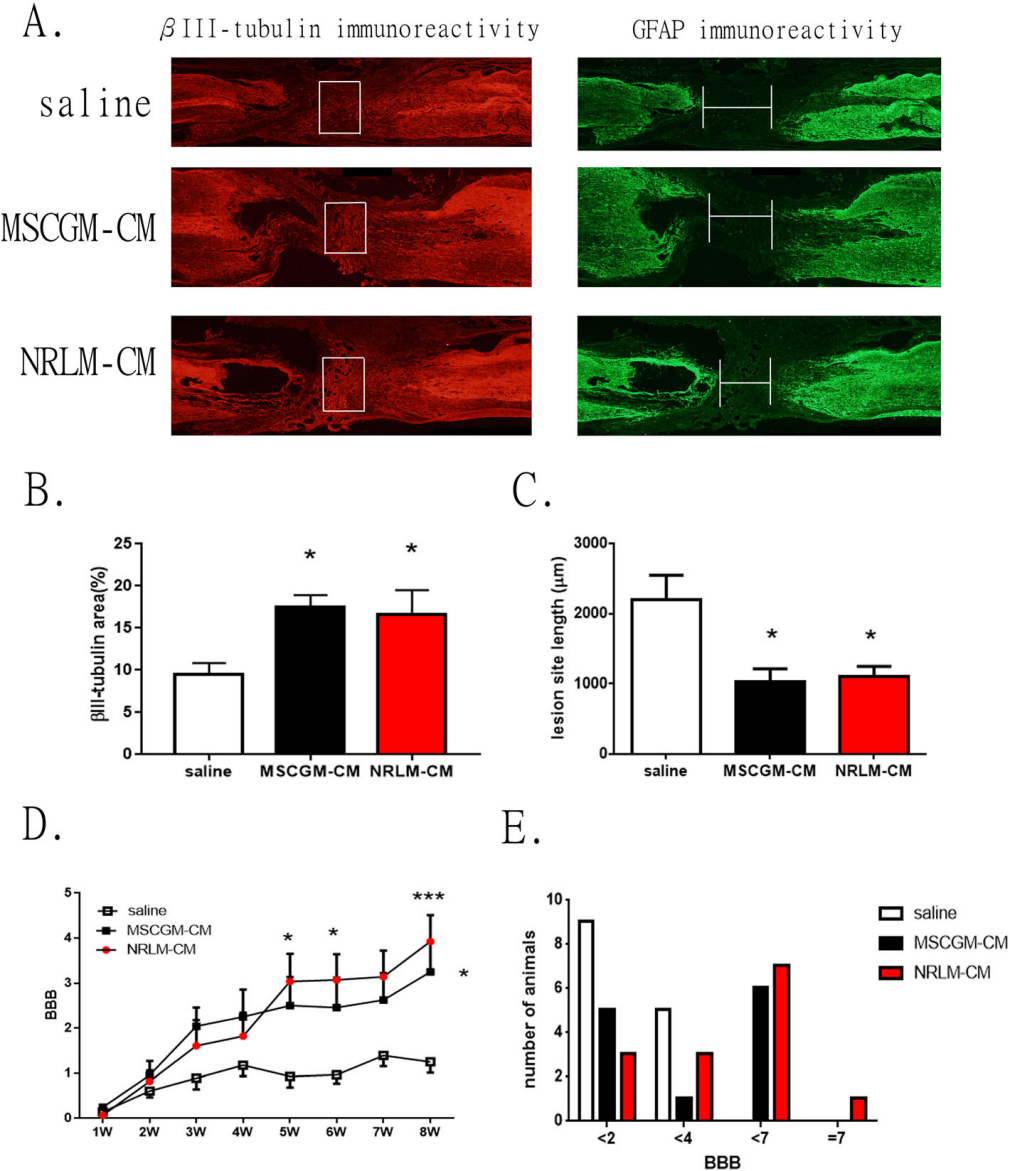
The beneficial effects of MSC-CM in spinal cord-injured rats in vivo. **a** The left side is rostral. Representative horizontal sections of the lesions showing anti-βIII-tubulin (to label nerve fibers) and anti-GFAP (to label astrocytes) immunostaining 2 months after transection and MSC-CM treatment. **b** Quantitative results of βIII-tubulin immunoreactivity (IR) are shown in the inset of **a**. **c** Quantitative results of GFAP IR-negative areas that define the length of the lesion as marked in the picture. Statistical significance was evaluated using one-way ANOVA with Tukey’s multiple comparisons test. *p < 0.05 and **p < 0.01 compared with the saline group. **d** The time course of BBB scores shows hindlimb improvement after the intrathecal administration of CM. The results are reported as the mean ± SEM. Statistical significance was evaluated using two-way ANOVA with Bonferroni multiple comparisons test. *p < 0.05 and ***p < 0.001 compared with the saline group. **e** A histogram showing the number of rats in each treatment group that met the listed BBB score thresholds at the eighth week of the behavioral test

The beneficial effect of MSC-CM was examined in complete spinal cord-transected rats in vivo. MSC-CM was administered to SCI rats through a pre-embedded intrathecal catheter. The first dose of CM was administered immediately after transection injury and then injected once a week for eight consecutive weeks. At the experimental endpoint, rat spinal tissues were collected and processed for immunohistochemical analysis. After SCI induction, cell debris and damaged axons were removed by macrophage phagocytosis, triggering astroglial activation, which led to the creation of an astroglial void region in the lesion. We thus investigated the neuronal and glial changes in the lesion area of the spinal cord after CM treatment. Double immunostaining was conducted to detect the immunoreactivity of βIII-tubulin (to detect nerve fibers) and GFAP (to detect astroglia) in the spinal cord sections of SCI rats; the results are shown in Fig. 2a. Figure 2b shows the quantified nerve fiber densities calculated from a square frame of Fig. 1a in the central GFAP-negative central. The number of βIII-tubulin-positive axons was significantly increased in the injury site in the CM treatment group compared to the control group (*p* < 0.05). Figure 2c shows the quantitation of the GFAP-negative area, defined as the lesion area, as marked in the spinal cord lesion. The length of the spinal cord lesion area was significantly shorter in both kinds of CM-treated spinal cords than in the saline-treated spinal cords (*p* < 0.05).

The results of the hindlimb behavioral evaluation in SCI rats are shown in Fig. 2d. Five weeks after injury, the hindlimb movement of SCI rats administered with NRLM-CM increased significantly compared to that of rats administered with saline (NRLM-CM BBB scores: fifth week, 3.1 ± 2.3; eighth week, 4.1 ± 2.2). Compared with that of the control, the BBB score of the MSCGM-CM group was significantly different until the eighth week (MSCGM-CM, 3.2 ± 2.2). We further analyzed the progress of the hindlimb behavior in each group of animals at 8 weeks postinjury (Fig. 2e). In the BBB score, a slight movement of three joints in the hindlimb is given a score of 4 points, while strong hindlimb movement is given a score of 7 points. No SCI rat in the control group had a score greater than 4 points, whereas the majority of the MSC-CM-treated rats had BBB scores of 4–7; one rat treated with NRLM-CM had a score of 7 points. These results indicate that NRLM-CM had an enhanced therapeutic effect.

### Clinical sample description

The above results show that a commercial line of human MSCs expanded in NRLM could produce beneficial CM. Next, we sought to examine whether NRLM could be used to expand MSCs from clinical bone marrow samples. Bone marrow samples were collected from 30 patients, aged 20–70 years old, who were receiving spinal fusion surgery. The average age of the patients was 49 years. There were 15 females and 15 males. Among the patients, 8 underwent C-spine fusion surgery, and 22 received L-spine fusion surgery. The detection of CD marker expression and adipocyte differentiation was used to identify all patient MSCs expanded in the 2 culture medium types. Detailed information of the clinical samples is shown in Additional file 1: Table S1.

### The characteristics of patient bone marrow MSCs cultured in NRLM and MSCGM

**Fig. 3:**
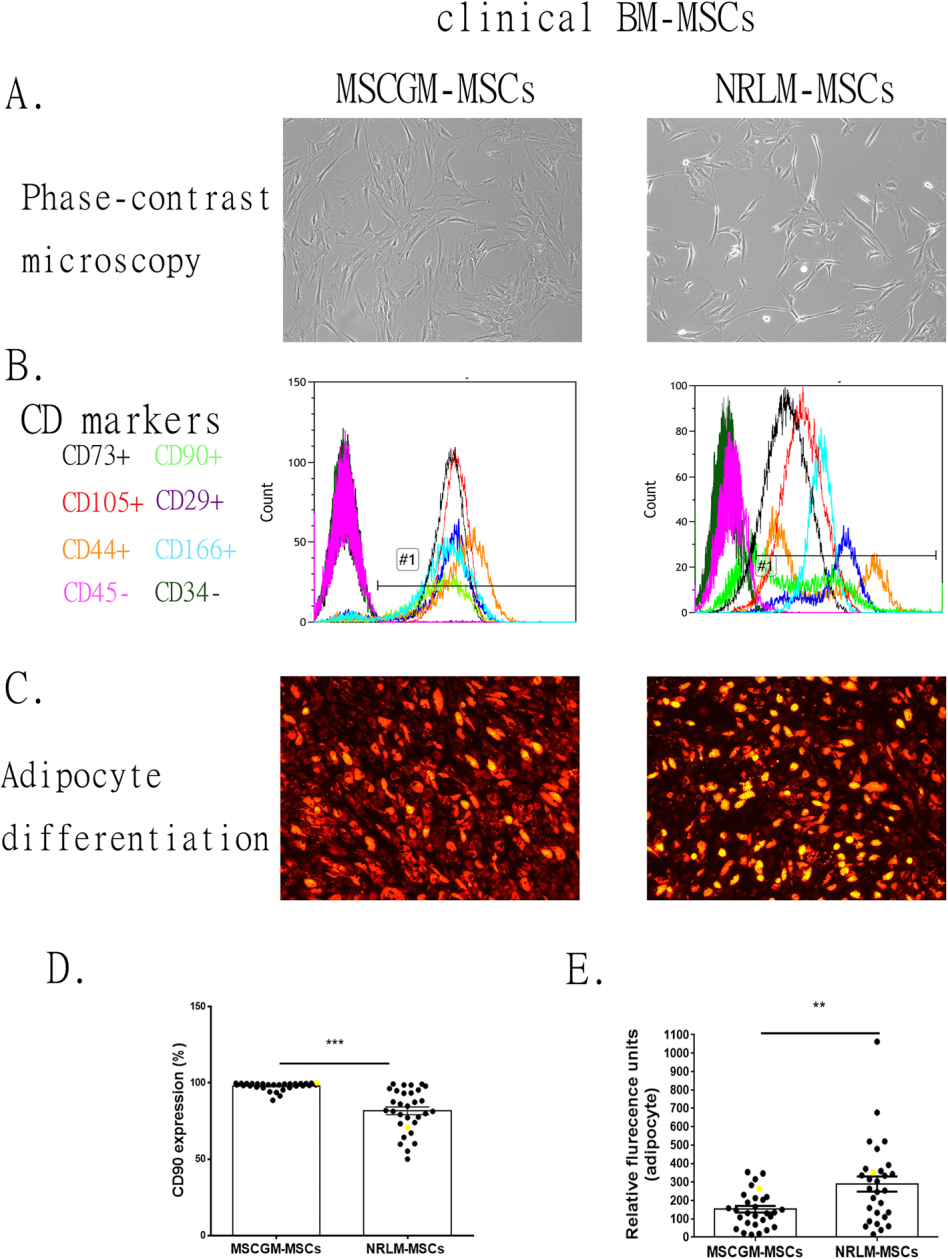
Characterization of clinical bone marrow MSCs expanded in MSCGM or NRLM. **a** Representative bright-field micrographs of clinical MSCs cultivated in MSCGM or NRLM. **b** Flow cytometry analysis of cell surface marker expression, including CD29, CD34, CD44, CD45, CD90, CD73, CD105, and CD166, in MSCGM- or NRLM-cultured MSCs. **c** Representative micrographs showing adipocyte differentiation potential (oil red-positive staining) from two kinds of cultivated MSCs. **d** Analysis of the CD90+ population in clinical MSCs cultivated in MSCGM or NRLM by flow cytometry (*N* = 31, including commercial MSC samples shown as yellow spots). Data are presented as the mean ± SEM. ***p < 0.001 by two-tailed *t* test. **e** MSC differentiation into adipocytes, which contain fluorescently labeled (red) oil. Bar chart showing the quantification of relative fluorescence units from 28 clinical MSC and commercial MSC samples (shown as yellow spots). Data are presented as the mean ± SEM. **p < 0.01 by two-tailed *t* test

The isolated bone marrow cells from patients were cultured in NRLM or MSCGM (MSCs cultured in NRLM are referred to as NRLM-MSCs, and those cultured in MSCGM are referred to as MSCGM-MSCs). Figure 3a shows that all cultured cells adhered to plastic dishes with fibroblast-like morphology. The cells expressed several MSC CD markers, including CD29, CD44, CD166, CD90, CD73, and CD105, but did not express CD34 and CD45 (Fig. 3b). The positive CD markers CD29, CD44, CD166, CD73, and CD105 were expressed by more than 90% of the MSCs (Additional file 2: Figure S1A). The CD90-expressing population was significantly smaller in NRLM-MSCs than in MSCGM-MSCs (Fig. 3d, 85.8% in NRLM-MSCs vs. 95.7% in MSCGM-MSCs, *p* < 0.001). The differentiation abilities, including adipogenesis, chondrogenesis, and osteogenesis, of MSCs cultured in MSCGM and NRLM were detected (Fig. 3c, Additional file 2: Figure S1B). The presence of fluorescently labeled oil in cells confirmed the potential of MSC to differentiate into adipocytes, and the adipocyte differentiation potential of NRLM-MSCs was greater than that of MSCGM-MSCs (Fig. 3e; *p* < 0.01).

### The proliferation activity of MSCs cultured in NRLM and MSCGM

**Fig. 4:**
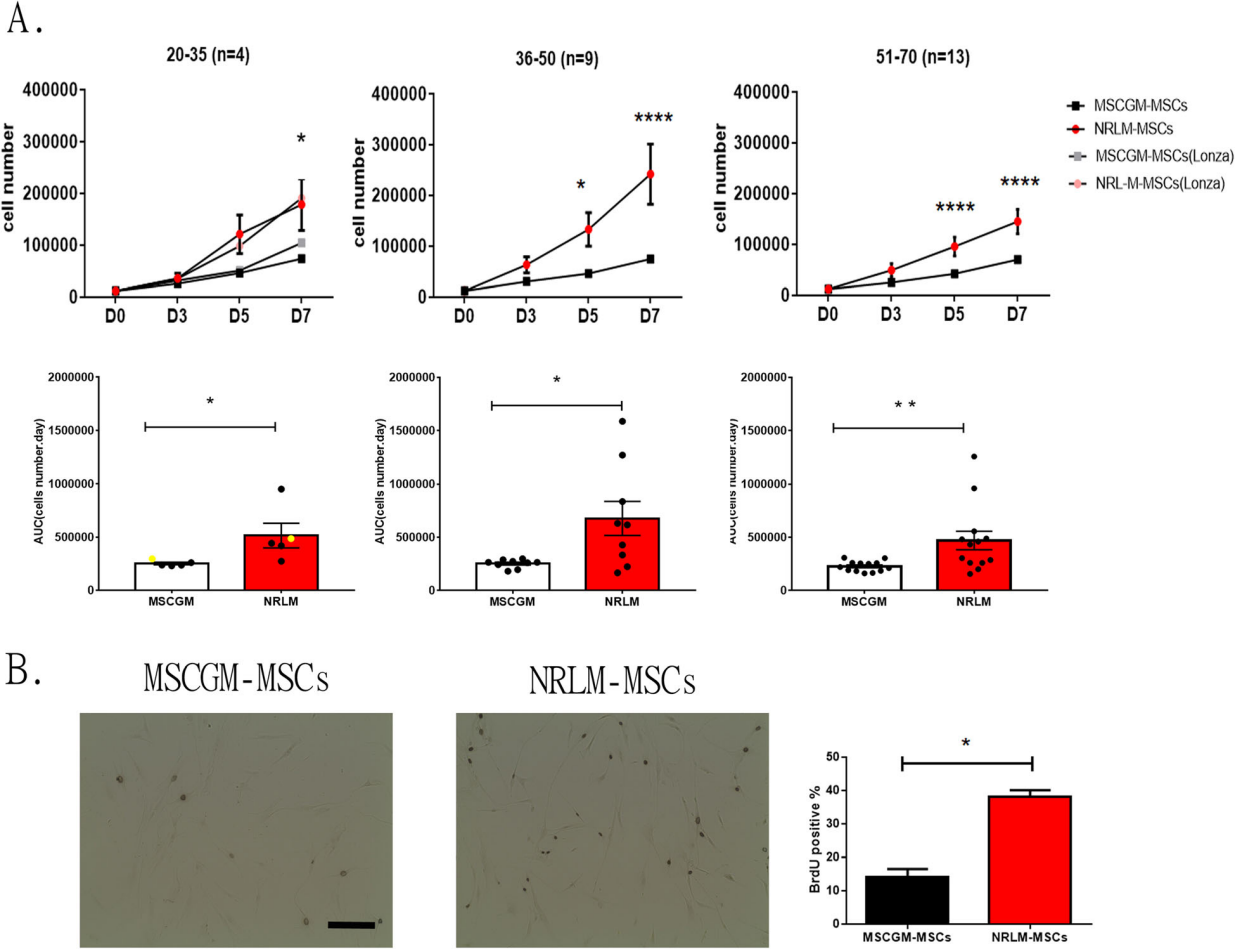
Comparison of the growth curves of clinical BM-MSCs cultured in MSCGM and NLRM. **a** The growth curves of the three age groups of MSCs were separately analyzed. The MSCs were seeded at a density of 1200 cells/well at the third passage and cultivated in two types of growth medium. The expanded cells were lifted and counted after 3, 5, and 7 days; the growth curves were plotted (upper panel), and the area under the growth curve was analyzed (lower panels). Quantification of the growth curve and area under the growth curve (AUC) showing that MSCs expanded faster in NLRM than MSCGM. Data of the AUC are the mean ± SEM. *p < 0.05 by two-tailed *t* test. The growth curve data were analyzed for statistical significance using two-way ANOVA with a Bonferroni multiple comparisons test (*p < 0.05 and ****p < 0.0001 compared with the saline group). **b** The proliferation of MSCs was analyzed by the incorporation of 25 μM BrdU for 6 h. Representative micrographs of BrdU-positive signals are shown. The BrdU(+) signal percentage per field was analyzed. Data are presented as the mean ± SEM. *p < 0.05 by two-tailed *t* test

For the ex vivo expansion of clinical MSCs, the NRLM was supplemented with the patients’ own plasma to avoid animal serum concerns. The growth of MSCs from clinical patient bone marrow in NRLM and MSCGM was evaluated. The MSCs of three age groups, 20–35 (young adult), 36–50 (middle-aged), and 51–70 (old age), were examined separately. At 3, 5, and 7 days after cell seeding, at a density of 1200 cells/well, in the two kinds of culture medium, the cell numbers were counted. The growth results of the commercial human MSCs (aged 21) were also included in the 20–35 age group of the clinical MSCs shown in Fig. 4a, as similar growth kinetics were found for the clinical and commercial MSCs. Compared with MSCGM, NRLM enhanced the expansion of MSCs and significantly increased the cell numbers in all age groups, even in the old age group, after 7 days (Fig. 4a, upper panel). The area under the growth curve analysis showed the same outcome. Furthermore, the number of BrdU-incorporated NRLM-MSCs was significantly greater than that of MSCGM-MSCs (Fig. 4b).

### The beneficial effect of clinical MSCs on neurological injury

**Fig. 5:**
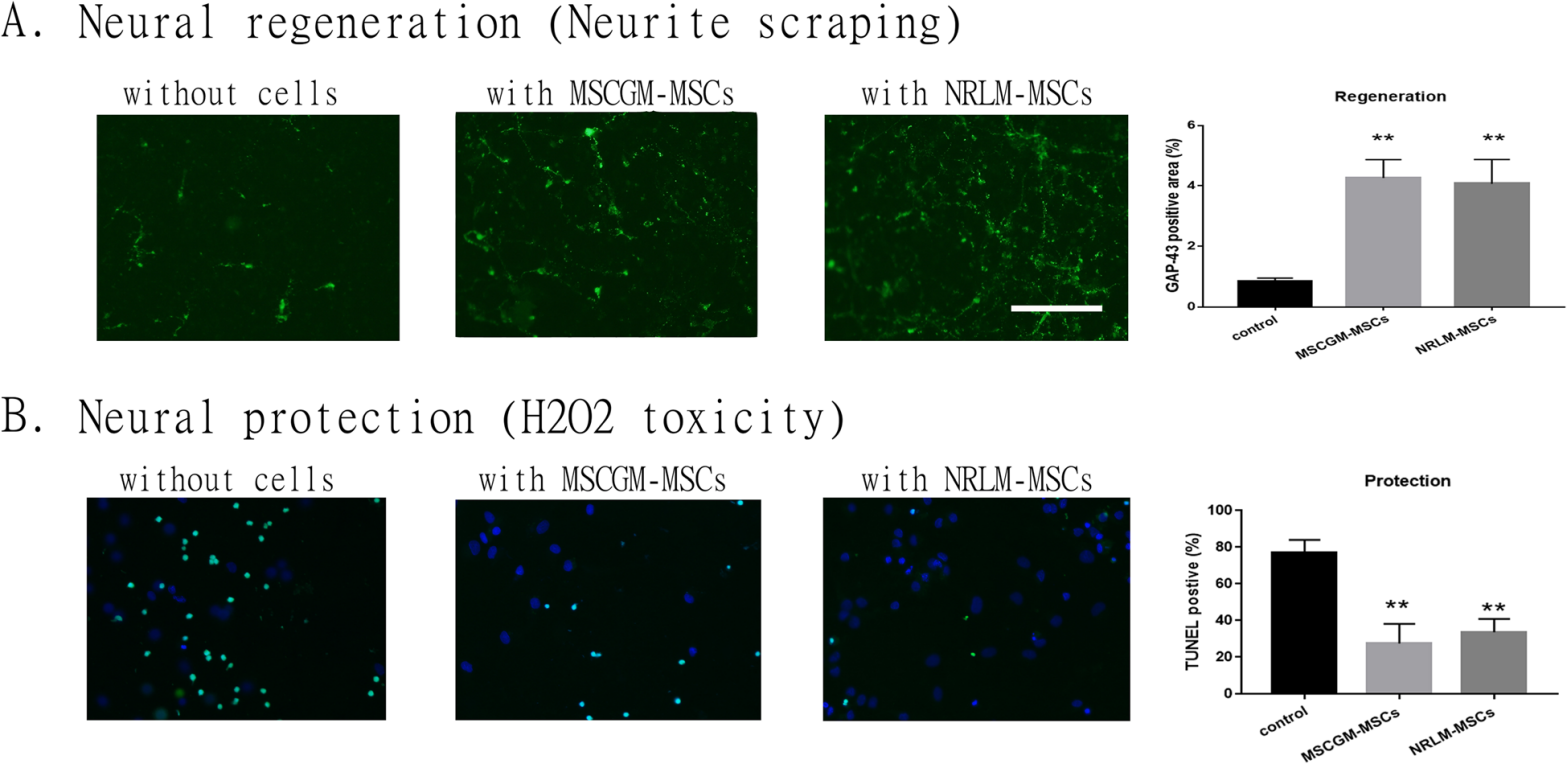
Analysis of the neuroregenerative and neuroprotective effects of clinical MSCs cultured in MSCGM or NLRM. **a** Neuroregeneration assay in spinal cord neuron (seeded in transwell inserts) and MSCs (seeded in culture wells) cocultures incubated for 5 days. The density of GAP-43-positive immunoreactivity in the bottom side of the transwell following scrape injury was analyzed and is shown in the bar chart. Representative GAP-43-positive micrographs are shown (bar = 200 μm). **b** Neuroprotection test in spinal cord neuron cultures and MSC cocultures after H_2_O_2_ (120 μM for one day) treatment. The apoptotic cells in neuron cultures after coculture with MSCs and H_2_O_2_ treatment were processed for TUNEL staining, and representative micrographs are shown (bar = 200 μm). The bar chart shows the quantification of the TUNEL-positive cells. Data are presented as the mean ± SEM. **p < 0.01 compared with the control group using one-way ANOVA with Tukey’s multiple comparisons test

Next, we examined and compared whether the clinical MSCs expanded in NRLM or MSCGM had neurological repair effects similar to those of the commercial MSCs. A coculture system with spinal cord neurons and MSCs was used to investigate the effect of the clinical MSCs. Figure 5a shows that after scratch injury, GAP-43-positive neurites in the spinal cord cultures extended through the transwell membrane in the presence or absence of MSCs. The GAP-43-positive area percentages were calculated by ImageJ software, and the quantitative results are shown in Fig. 5b. The GAP-43-positive area percentages were significantly increased in the MSC coculture group (*p* < 0.01).

Oxidative stress was induced in cells after SCI. We treated spinal cord neuron cultures or cocultures with H_2_O_2_ to mimic oxidative stress and detected apoptotic cells by TUNEL staining, as shown in Fig. 5c. Figure 5d shows the quantification of the TUNNEL-positive cells in the spinal cord neuron cultures. After treatment with H_2_O_2_ for 24 h, approximately 77% of the cells in the spinal neuron culture were apoptotic. MSC coculture significantly decreased the percentage of TUNNEL-positive (apoptotic) cells (*p* < 0.01). Both kinds of CM from the two cultures showed similar neuroprotective/neuroregenerative efficacy.

### The clinical MSC secretome

**Fig. 6:**
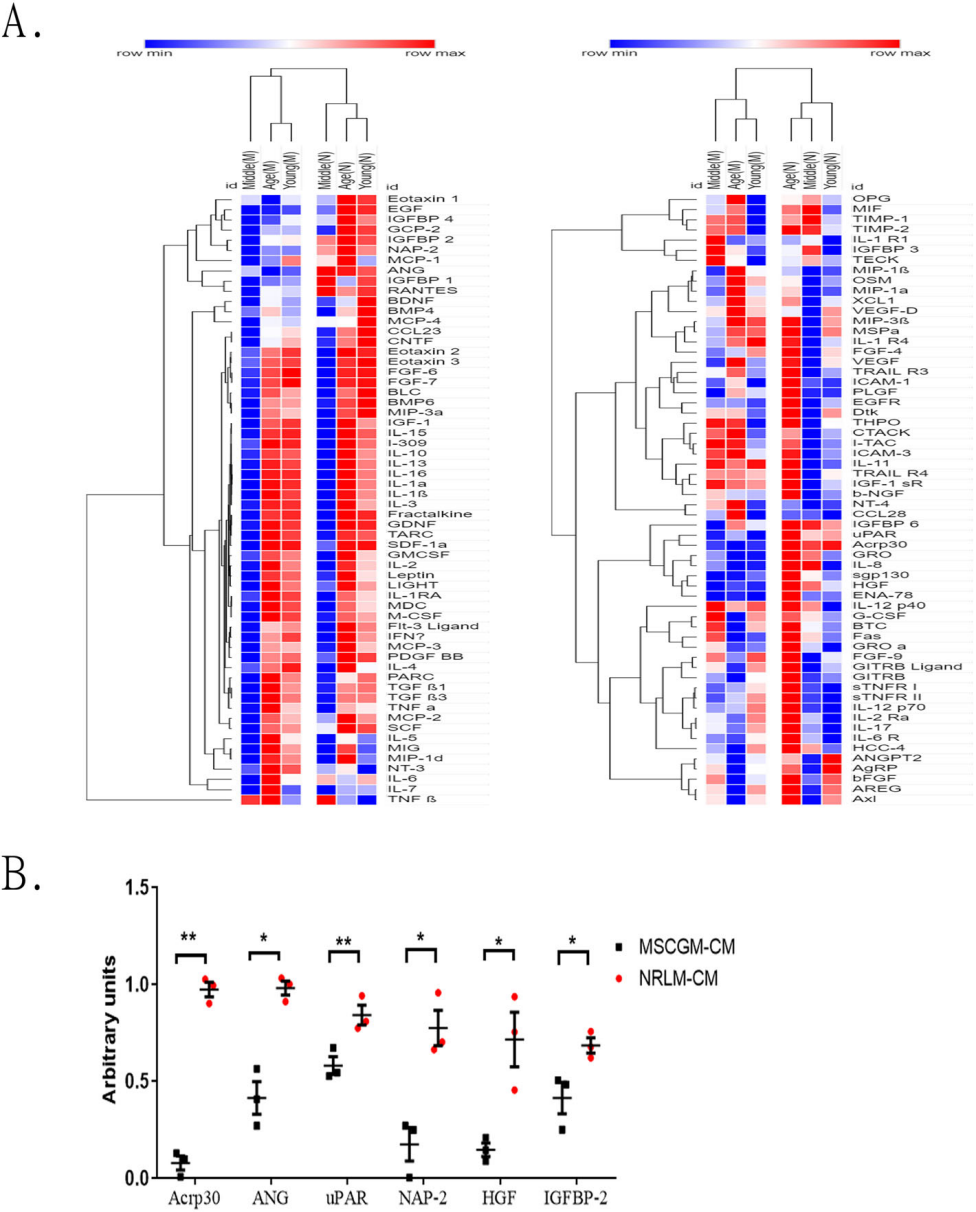
Characterization of the CM derived from 2 kinds of cultivated MSCs, grouped by donor age and by cytokine array analysis. **a** Hierarchical clustering of the rows and columns was carried out according to the cytokine array intensity values (calculated by ImageJ software) for MSCGM-CM (M) and NRLM-CM (N) samples from the different patients (young, middle-aged, and older) using MORPHEUS one minus Pearson correlation (Versatile matrix visualization and analysis software; https://software.broadinstitute.org/morpheus). **b** The statistical graph shows that the levels of 6 out of 120 proteins/cytokines were significantly different between the 2 kinds of cultivated MSC-CM. A human cytokine antibody array kit from RayBiotech was used to analyze the MSC-CM, revealing the presence of 120 cytokines and trophic factors. Data are presented as the mean ± SEM. *p < 0.05 and **p < 0.01 by two-tailed *t* test (*N* = 3)

The above neuroprotective/neuroregenerative effects of human MSCs, either from commercial or clinical isolates, were assessed in a coculture study. The beneficial effects of the MSC-CM were also demonstrated in SCI rats in vivo. These experiments indicated that the effects are mainly derived from the factors released by MSCs, namely, the secretome. It would be interesting to identify the differences in the MSC-CM from different patient bone marrow samples. Thus, 2 MSC-CM samples from 3 patients (aged 20, 39, and 53) were collected and individually analyzed by human cytokine array. The expression ratio of 120 proteins/cytokines in the MSC secretome is shown as a histogram in Additional file 3: Figure S2. We found that the expression levels of monocyte chemotactic protein-1/CCL2 (MCP-1), *osteoprotegerin (*OPG), and tissue inhibitor of metalloproteinase-1 (TIMP-1) were highly consistent in all different conditioned media. The relative expression of 120 proteins from the NRLM-CM and MSCGM-CM of a single patient’s MSCs per age group was calculated and shown in a hierarchical clustering diagram (Fig. 6a). The cytokine results of the NRLM-CM and MSCGM-CM from the MSCs of 3 patients were further compared. The levels of 6 out of 120 factors, including adiponectin (Acrp30), *angiogenin (*ANG), hepatocyte growth factor (HGF), neutrophil-activating peptide 2 (*NAP*-*2*), urokinase-type plasminogen activator receptor (uPAR), and insulin growth factor-binding protein 2 (*IGFBP-2*), were significantly increased in the NRLM-CM compared with the MSCGM-CM (Fig. 6B).

## Discussion

In this study, we developed a culture medium, NRLM, for clinical hMSC expansion, producing enhanced yields and therapeutic effects. A commercial hMSC line was first used to establish the expansion conditions and efficacy in cultures as well as in SCI rats. Furthermore, the growth of clinical hMSCs, cell characteristics, and differentiation capacity in NRLM were tested and compared to those of cells grown in conventional control medium (referred to as MSCGM); NRLM increased the fold expansion of MSCs. The clinical MSC-CM displayed effective neurological repair effects. Importantly, NRLM also markedly elevated the growth rate of MSCs from older donors.

The beneficial therapeutic effects are crucial for the application of MSCs in SCI. There is evidence that the anti-apoptotic and anti-inflammatory effects of MSC transplantation are derived from paracrine mechanisms [28, 40]. Recent studies have further demonstrated that MSC-CM improves hindlimb motor function after SCI [28, 30, 41]. Although MSC-CM has not been used in clinical treatment, its potential for future clinical applications seems promising. CM is xeno- and serum-free and, therefore, raises no cell survival concerns after in vivo treatment. In this study, we found that two conditioned media derived from different MSC cultures promoted neurite outgrowth and inhibited LPS stimulation, with the NRLM-CM having the greatest effect (Fig. 1b, c). We further examined the therapeutic effects of CM in a severe injury model of completely transected SCI in rats. The in vivo results showed that MSC-CM effectively improved hindlimb function restoration after SCI in rats, with the NRLM-CM being the most effective (Fig. 2). Consistently, the neurite extension effect of MSCs in neuron-MSC cocultures confirmed the benefits of the MSC secretome for neural regeneration (Fig. 5).

In addition, the yield and safety are the other issues for MSC clinical applications. Some commercial serum-free media designed for MSC culture avoid the use of animal sera but still use attachment substrates from human tissue extracts for initial MSC adhesion [42], suggesting a risk of human pathogen contamination. We previously used serum-free and bFGF/EGF-supplemented medium for MSC culture and showed that MSCs could be maintained for expansion but not for initial seeding [31]. The NRLM supplemented with bFGF, EGF, and the patients’ own plasma enhanced the initial seeding of MSCs from the bone marrow. Then, we tested the growth of clinical hMSCs, cell characteristics, and differentiation capacity of cells grown in NRLM and compared them to those of cells grown in a conventional medium. The findings showed that NRLM enhances MSC expansion. Although the effects of bFGF and EGF on MSC proliferation have been well demonstrated by other groups [33–36, 43, 44], we still grouped the MSCs by donor age to investigate the effect of NRLM on MSC growth at different ages. Our data demonstrated that even in the older age group, NRLM significantly enhanced the growth of MSCs, which was not different from that of the younger group (Fig. 4a). This medium could reduce the required amount of MSCs and manufacturing time and accelerate the production of MSCs in therapeutically relevant numbers.

EGF and bFGF have been shown to improve the adipocyte differentiation capacity of MSCs [43, 45, 46], and bFGF addition during the expansion of hMSCs decreases the expression of the cell marker CD90 [35, 44]. We previously showed that MSCs maintained in serum-free and bFGF/EGF-supplemented medium have low CD90 expression levels [31]. Furthermore, in this study, the CD90-positive population of NRLM-expanded hMSCs was reduced in both commercial and clinical samples. Therefore, we hypothesized that the low CD90 expression in NRLM-MSCs could be due to the growth factors. In addition to its effect on CD90, our results also showed that NRLM enhanced the adipocyte differentiation potential of hMSCs (Fig. 3e). The expression of CD90 gradually decreases during adipogenesis, and mature adipocytes express almost no CD90. The reduction of CD90 expression by siRNA treatment in MSCs has been shown to enhance MSC adipogenic differentiation [47], whereas methylation inhibitor treatment to maintain CD90 expression during adipogenic differentiation reduces adipocyte formation [48]. This finding is consistent with the characteristics presented by our NRLM-MSCs from both clinical patients and commercial MSCs. Therefore, we suggest that the effect of NRLM on proliferation, CD90 expression, and adipogenic differentiation in MSCs might induce the production of a superior CM for nerve repair.

The MSC-CM generally contains angiogenic factors, trophic factors, chemokines, proinflammatory cytokines, anti-inflammatory cytokines, etc. [17]. We conducted a cytokine array using MSCGM-CM, MSC-CM from a clinical patient, and NRLM-CM from the three age groups. Notably, in our previous study, the expression of TIMP-1 by MSCs cultivated in serum-free and bFGF/EGF-supplemented medium was reduced, which influenced the ability of MSCs to inhibit inflammation [31]. However, in this study, the addition of FBS/donor plasma preserved TIMP-1 expression, which was not different from that in cells cultured in a standard medium (Additional file 3: Figure S2). Surprisingly, there was a considerable difference in the adiponectin level between the two conditioned media, which was tenfold higher in the NRLM-CM than in the MSCGM-CM (Fig. 6b). Adiponectin is expressed in adipocyte and adipocyte-derived mesenchymal stromal cells. High adiponectin expression induces adipocyte differentiation [49]. The high adiponectin expression result is consistent with the high adipogenesis capacity of NRLM-MSCs. Adiponectin receptors are expressed in the central nervous system, but adiponectin is not. The intrathecal administration of adiponectin attenuates thermal hyperalgesia and mechanical hypersensitivity and reduces inflammation [50]. ANG is an angiogenic factor, and the loss of ANG function is involved in neurodegenerative diseases, such as amyotrophic lateral sclerosis (ALS) [51, 52]. The use of an adenovirus to express ANG can effectively improve the function of the hindlimbs after spinal injury [53]. HGF has also been found to promote functional recovery in rats after SCI [54]. Therefore, our study results suggest that the observed superiority of NRLM-CM over MSCGM-CM may result from the aforementioned secreted factors.

## Conclusions

Overall, our study results demonstrate the NRLM culture system enhances MSC expansion and produces an enhanced CM for SCI repair. Therefore, NRLM is the preferred medium for the large-scale production of functionally competent MSCs for future clinical applications.

## Supplementary information

**Additional file 1: Table S1.** The list for BM-MSC from clinical patients.

**Additional file 2: Figure S1.** Characterization of clinical/commercial BM-MSCs expanded in MSCGM or NLRM. (A) The bar chart shows the percentage of cell surface marker expression in MSCGM- or NRLM-expanded MSCs; the dotted line indicates 90%. (B) MSCs were cultured in chondrogenesis medium for 14 days, and alcian blue staining was used to detect matrix proteoglycan. MSCs were cultured in osteogenesis medium for 10 days, and the expression of alkaline phosphatase was detected by alkaline phosphatase substrate (Blue AP Substrate Kit SK-5300, Vector).

**Additional file 3: Figure S2.** The expression levels of 120 proteins in the CM of BM-MSCs by cytokine array analysis. Bar diagrams represent the ratio of the mean spot pixel density/positive-control spot pixel density. Antibody arrays were performed on two types of MSC-CM from each of three patients. The results are presented as the mean ± SEM.

## Data Availability

Please contact the corresponding author for data requests.

## Abbreviations

MSCs: Mesenchymal stem cells
MSC-CM: Mesenchymal stem cell-derived conditioned medium
NRLM-MSCs: MSCs cultivated in the neural regeneration laboratory medium
MSCGM-MSCs: MSCs cultivated in the standard MSC growth medium
OGD: Oxygen-glucose deprivation
ANG: Angiogenin
HGF: Hepatocyte growth factor
NAP-2: Neutrophil-activating peptide 2
uPAR: Urokinase-type plasminogen activator receptor
IGFBP-2: Insulin-like growth factor-binding protein 2
ALS: Amyotrophic lateral sclerosis
