# Supplementary material for: The superiority of conditioned medium derived from rapidly expanded mesenchymal stem cells for neural repair
Source: Stem Cell Res Ther. 2019 Dec 16;10:390. doi: 10.1186/s13287-019-1491-7 (PMC6916259; doi:10.1186/s13287-019-1491-7)
Supplement: Supplementary file 1 — Additional file 1: Table S1. The list for BM-MSC from clinical patients. [file 13287_2019_1491_MOESM1_ESM.docx]

| Table S1: The list for BM-MSC from clinical patients | | | | | |  | |
| --- | --- | --- | --- | --- | --- | --- | --- |
| Number | Age | Gender | Spine | Culture medium | Application of this study | |  |
| NRL 01 | 62 | F | L | MSCGM | F,AD,GC | |  |
|  |  |  |  | NRLM | F,AD,GC | |  |
| NRL 02 | 48 | M | L | MSCGM | F,AD,GC, axon regeneration | |  |
|  |  |  |  | NRLM | F,AD,GC, axon regeneration | |  |
| NRL 03 | 68 | M | L | MSCGM | F,AD,GC | |  |
|  |  |  |  | NRLM | F,AD,GC | |  |
| NRL 04 | 28 | M | L | MSCGM | F,AD,GC, axon regeneration | |  |
|  |  |  |  | NRLM | F,AD,GC, axon regeneration | |  |
| NRL 05 | 51 | F | L | MSCGM | F,AD,GC, protection | |  |
|  |  |  |  | NRLM | F,AD,GC, protection | |  |
| NRL 06 | 33 | M | L | MSCGM | F,AD,GC, protection | |  |
|  |  |  |  | NRLM | F,AD,GC, protection | |  |
| NRL 07 | 55 | F | L | MSCGM | F,AD,GC | |  |
|  |  |  |  | NRLM | F,AD,GC | |  |
| NRL 08 | 56 | M | L | MSCGM | F,AD,GC | |  |
|  |  |  |  | NRLM | F,AD,GC | |  |
| NRL 09 | 61 | M | L | MSCGM | F,AD,GC | |  |
|  |  |  |  | NRLM | F,AD,GC | |  |
| NRL 10 | 41 | M | L | MSCGM | F,AD,GC | |  |
|  |  |  |  | NRLM | F,AD,GC | |  |
| NRL 11 | 49 | F | C | MSCGM | F,AD,GC | |  |
|  |  |  |  | NRLM | F,AD,GC | |  |
| NRL 12 | 63 | M | L | MSCGM | F,AD,GC | |  |
|  |  |  |  | NRLM | F,AD,GC | |  |
| NRL 13 | 59 | F | C | MSCGM | F,AD,GC | |  |
|  |  |  |  | NRLM | F,AD,GC | |  |
| NRL 14 | 46 | F | C | MSCGM | F | |  |
|  |  |  |  | NRLM | F,AD | |  |
| NRL 15 | 39 | F | C | MSCGM | F,AD,GC, array | |  |
|  |  |  |  | NRLM | F,AD,GC, array | |  |
| NRL 16 | 52 | M | C | MSCGM | F,AD | |  |
|  |  |  |  | NRLM | F,AD,GC | |  |
| NRL 17 | 53 | F | C | MSCGM | F,AD | |  |
|  |  |  |  | NRLM | F,AD,GC | |  |
| NRL 18 | 41 | F | C | MSCGM | F,AD,GC | |  |
|  |  |  |  | NRLM | F,AD,GC | |  |
| NRL 19 | 36 | M | L | MSCGM | F,AD,GC | |  |
|  |  |  |  | NRLM | F,AD,GC | |  |
| NRL 20 | 48 | M | C | MSCGM | F,AD,GC, BrdU | |  |
|  |  |  |  | NRLM | F,AD,GC, BrdU | |  |
| NRL 21 | 58 | M | L | MSCGM | F,AD,GC | |  |
|  |  |  |  | NRLM | F,AD,GC | |  |
| NRL 22 | 58 | F | L | MSCGM | F,AD,GC | |  |
|  |  |  |  | NRLM | F,AD,GC | |  |
| NRL 23 | 54 | F | L | MSCGM | F,AD,GC | |  |
|  |  |  |  | NRLM | F,AD,GC | |  |
| NRL 24 | 60 | M | L | MSCGM | F,AD,GC | |  |
|  |  |  |  | NRLM | F,AD,GC | |  |
| NRL 25 | 50 | F | L | MSCGM | F,AD,GC | |  |
|  |  |  |  | NRLM | F,AD,GC | |  |
| NRL 26 | 50 | F | L | MSCGM | F,AD,GC, axon regeneration | |  |
|  |  |  |  | NRLM | F,AD,GC, axon regeneration | |  |
| NRL 27 | 53 | M | L | MSCGM | F,AD,GC,array | |  |
|  |  |  |  | NRLM | F,AD,GC,array | |  |
| NRL 28 | 20 | M | L | MSCGM | F,AD,GC, array | |  |
|  |  |  |  | NRLM | F,AD,GC, array | |  |
| NRL 29 | 31 | F | L | MSCGM | F,AD,GC | |  |
|  |  |  |  | NRLM | F,AD,GC | |  |
| NRL 30 | 47 | F | L | MSCGM | F | |  |
|  |  |  |  | NRLM | F,AD | |  |
