# Supplementary material for: The superiority of conditioned medium derived from rapidly expanded mesenchymal stem cells for neural repair
Source: Stem Cell Res Ther. 2019 Dec 16;10:390. doi: 10.1186/s13287-019-1491-7 (PMC6916259; doi:10.1186/s13287-019-1491-7)
Supplement: Supplementary file 2 — Additional file 2: Figure S1. Characterization of clinical/commercial BM-MSCs expanded in MSCGM or NLRM. (A) The bar chart shows the percentage of cell surface marker expression in MSCGM- or NRLM-expanded MSCs; the dotted line indicates 90%. (B) MSCs were cultured in chondrogenesis medium for 14 days, and alcian blue staining was used to detect matrix proteoglycan. MSCs were cultured in osteogenesis medium for 10 days, and the expression of alkaline phosphatase was detected by alkaline phosphatase substrate (Blue AP Substrate Kit SK-5300, Vector). [file 13287_2019_1491_MOESM2_ESM.docx]

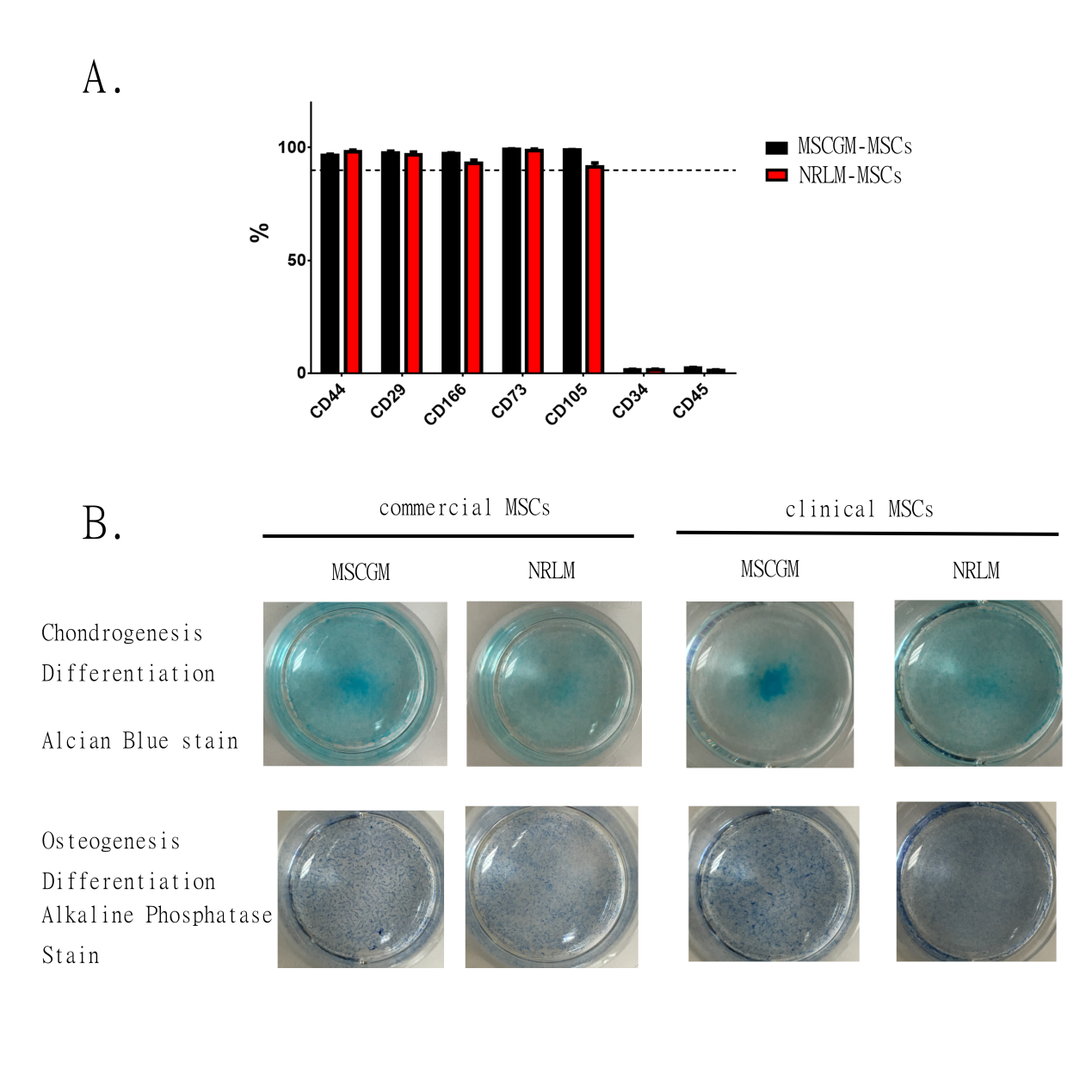
**Figure S1:** **Characterization of clinical/commercial BM-MSCs expanded in MSCGM or NLRM.** (A) The bar chart shows the percentage of cell surface marker expression in MSCGM- or NRLM-expanded MSCs; the dotted line indicates 90%. (B) MSCs were cultured in chondrogenesis medium for 14 days, and alcian blue staining was used to detect matrix proteoglycan. MSCs were cultured in osteogenesis medium for 10 days, and the expression of alkaline phosphatase was detected by alkaline phosphatase substrate (Blue AP Substrate Kit SK-5300, Vector).
