# Supplementary material for: The superiority of conditioned medium derived from rapidly expanded mesenchymal stem cells for neural repair
Source: Stem Cell Res Ther. 2019 Dec 16;10:390. doi: 10.1186/s13287-019-1491-7 (PMC6916259; doi:10.1186/s13287-019-1491-7)
Supplement: Supplementary file 3 — Additional file 3: Figure S2. The expression levels of 120 proteins in the CM of BM-MSCs by cytokine array analysis. Bar diagrams represent the ratio of the mean spot pixel density/positive-control spot pixel density. Antibody arrays were performed on two types of MSC-CM from each of three patients. The results are presented as the mean ± SEM. [file 13287_2019_1491_MOESM3_ESM.docx]

**
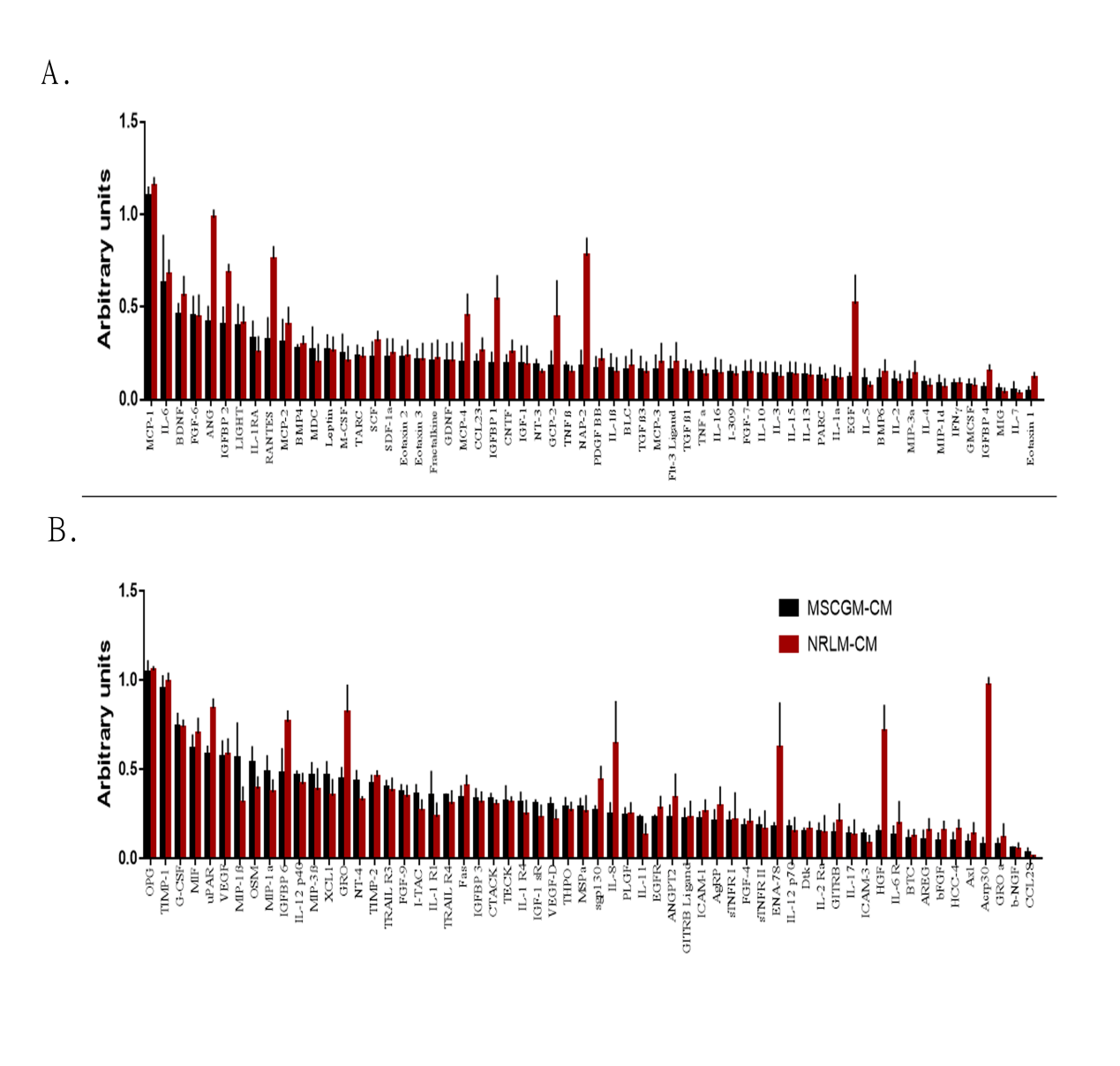
Figure S2:** **The expression levels of 120 proteins in the CM of BM-MSCs by cytokine array analysis.** Bar diagrams represent the ratio of the mean spot pixel density/positive-control spot pixel density. Antibody arrays were performed on two types of MSC-CM from each of three patients. The results are presented as the mean ± SEM.
